# A Rare Presentation of Wernicke’s Encephalopathy With Internuclear Ophthalmoplegia

**DOI:** 10.7759/cureus.86321

**Published:** 2025-06-18

**Authors:** Montasir Elmobark, Osama Ahmed, Wisam Ali

**Affiliations:** 1 Gastroenterology, University Hospital of North Tees, Stockton, GBR; 2 General Internal Medicine, University Hospital of North Durham, Durham , GBR; 3 Emergency Department, Best Care Hospital, Khartoum, SDN

**Keywords:** fibrous dysplasia (fd), gait ataxia, internuclear ophthalmoplegia, thiamine or vitamin b1 deficiency, wernicke’s encephalopathy (we)

## Abstract

Wernicke’s encephalopathy (WE) is a neurological emergency caused by thiamine (vitamin B1) deficiency and classically presents with a triad of confusion, ophthalmoplegia, and ataxia. Although classically associated with chronic alcohol misuse, it may also occur in non-alcoholic individuals due to causes such as persistent vomiting, malnutrition, or gastrointestinal disorders. We present a rare case of WE in a 35-year-old woman who developed internuclear ophthalmoplegia (INO), a highly unusual manifestation in this context. She presented with a one-week history of blurred and double vision, gait unsteadiness, and persistent bilious vomiting, resulting in significant weight loss. Neurological examination revealed right INO with preserved convergence, positive Romberg’s sign, and gait ataxia. Imaging revealed fibrous dysplasia of the left temporal bone, but no intracranial abnormalities were suggestive of WE. Based on clinical findings, a clinical diagnosis of WE secondary to persistent vomiting was made. Prompt intravenous thiamine led to a rapid resolution of symptoms. This case underscores the importance of early recognition of atypical WE manifestations, even in the absence of classic imaging findings. Timely thiamine replacement is critical, as delayed or inadequate treatment can result in up to 20% mortality, with approximately 85% of survivors developing Korsakoff’s psychosis.

## Introduction

Wernicke’s encephalopathy (WE) is a neurological emergency caused by thiamine (vitamin B1) deficiency. It typically presents with a triad of ophthalmoplegia, ataxia, and confusion [1]. Although WE is classically associated with chronic alcohol misuse, non-alcoholic causes such as prolonged vomiting, gastrointestinal disorders, and malnutrition are also well-established triggers [2].

Internuclear ophthalmoplegia (INO) is a rare and atypical ocular finding in WE. INO is more commonly seen in conditions such as multiple sclerosis in younger patients and brainstem stroke in the elderly. It results from lesions in the medial longitudinal fasciculus (MLF), a key neural tract involved in coordinating horizontal gaze [3,4]. Its presence in WE is uncommon and may confound the diagnosis, especially when the classic triad is incomplete or imaging is non-diagnostic.

Early recognition of WE and immediate high-dose thiamine treatment are crucial. If the condition is missed or inappropriately treated with insufficient thiamine, outcomes are often severe. Mortality rates in such cases approach 20%, and among survivors, nearly 85% may develop Korsakoff’s psychosis - a chronic syndrome marked by profound memory impairment, disorientation, and confabulation [5].

We present a rare case of WE in a young woman with persistent vomiting and INO, who was successfully treated with prompt high-dose thiamine therapy. This case emphasizes the importance of clinical vigilance in identifying atypical presentations of WE and initiating immediate intervention to prevent permanent neurological sequelae.

## Case presentation

A 35-year-old woman with a history of gastroesophageal reflux disease presented with a one-week history of progressively worsening blurred and double vision, unsteadiness while walking, and frequent bilious vomiting. These symptoms began following an oesophago-gastro-duodenoscopy (OGD). She also reported mild headaches and had lost approximately 4 kg over the preceding month.

On neurological examination, her pupils were equal and reactive to light with no evidence of ptosis. The left eye tracked normally on lateral gaze, but the right eye failed to adduct, indicating right INO. Convergence remained intact, and bilateral horizontal nystagmus was noted. Muscle tone and strength were normal in all extremities. Deep tendon reflexes were intact. Coordination testing revealed mild dysmetria, and she had a positive Romberg’s test. Her gait was broad-based and ataxic.

**Figure 1:**
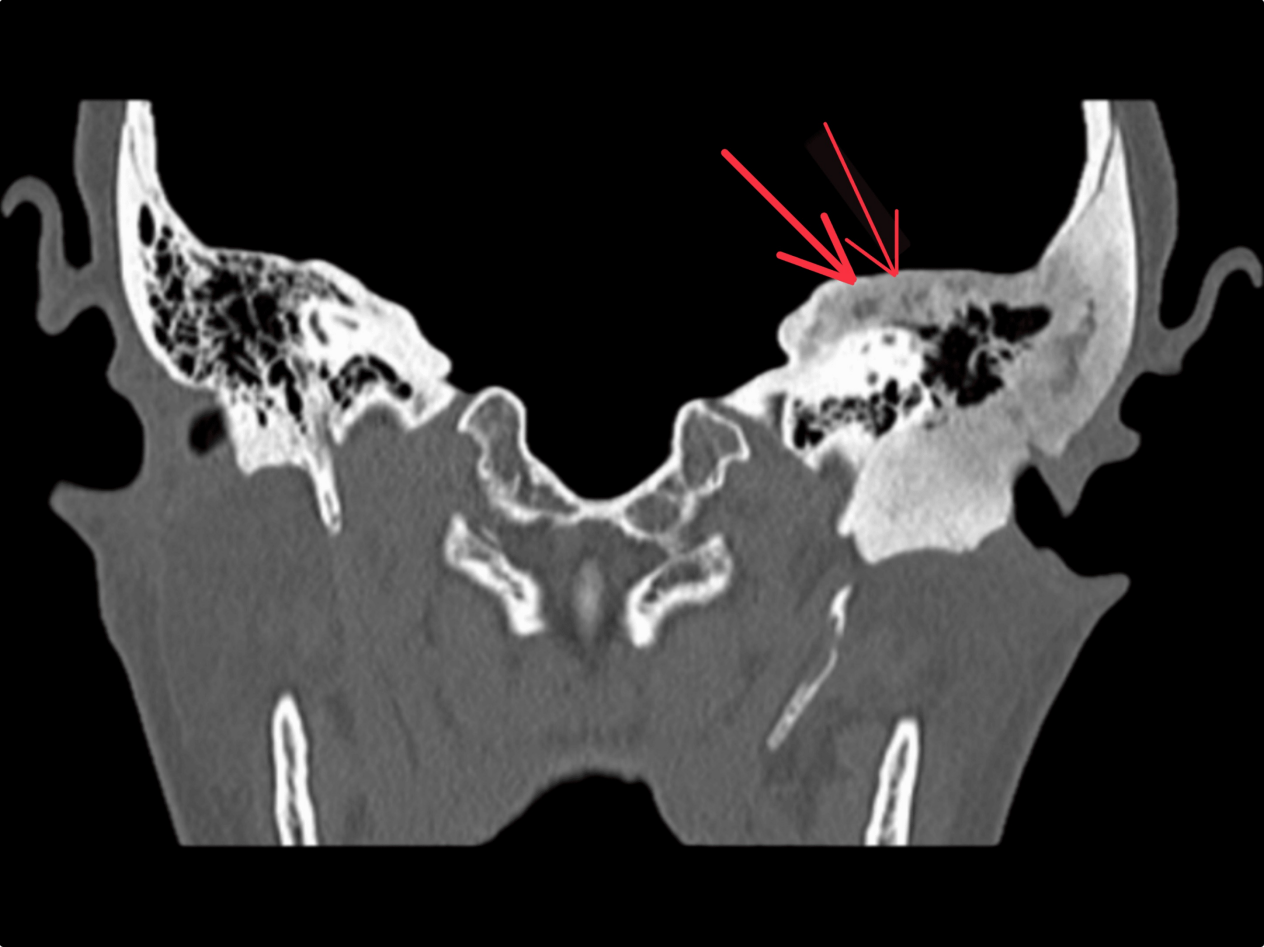
CT Temporal Bones, Coronal View Coronal CT of the temporal bones showing fibrous dysplasia in the petrous and squamous portions of the left temporal bones (red arrows).

**Figure 2:**
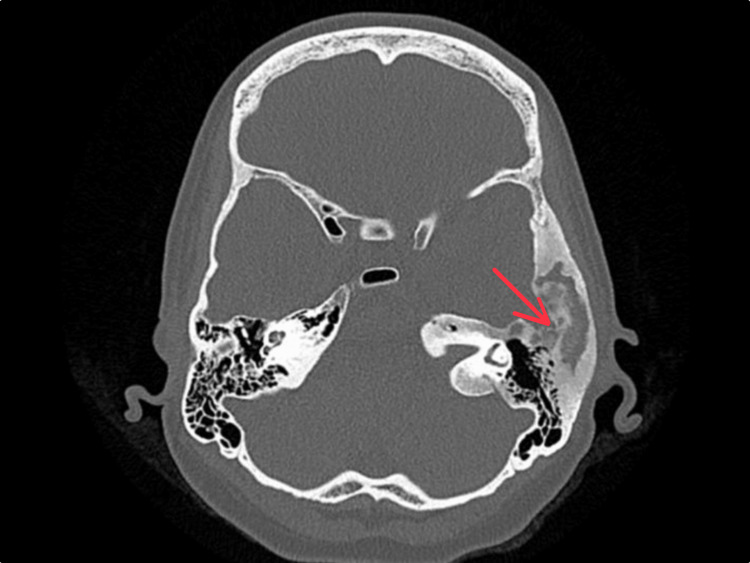
CT Temporal Bones, Axial View Axial CT of the temporal bone demonstrating the characteristic ground-glass appearance of fibrous dysplasia in the left temporal bone (red arrow).

A CT scan of the brain showed no acute intracranial abnormalities. However, a subsequent CT of the temporal bones revealed fibrous dysplasia involving the petrous and squamous portions of the left temporal bone (Figures 1-2). An MRI of the brain showed no signal changes consistent with WE and no evidence of demyelination or structural lesions

Given the clinical picture and risk factors, a diagnosis of WE secondary to thiamine deficiency from prolonged vomiting was made. She was immediately started on intravenous Pabrinex (a concentrated vitamin B complex preparation) administered three times daily for five days. This was followed by oral supplementation with thiamine, folate, and vitamin B12. A multidisciplinary team-including neurology, ophthalmology, and ENT-was involved in her management.

The patient showed significant improvement in her symptoms. By day six, her vision was near normal, and her gait had returned to baseline. She was discharged with instructions for continued oral supplementation and dietary modifications.

At a one-month follow-up, she reported complete resolution of symptoms. A neuro-ophthalmologic evaluation confirmed near-normal ocular motility, and there were no residual neurological deficits.

## Discussion

This case presents a rare occurrence of INO in the setting of WE. INO is typically due to a lesion in the MLF and is more commonly associated with multiple sclerosis in younger patients and ischemic strokes in the elderly [3,4]. Its appearance in the setting of thiamine deficiency is unusual and could lead to misdiagnosis if not considered carefully.

Neuroimaging is a useful adjunct but is not definitive in diagnosing WE. Typical MRI findings include symmetric hyperintensities in the periaqueductal area, mammillary bodies, and medial thalami, particularly in alcoholic patients [6]. However, imaging in non-alcoholic WE may be unremarkable, emphasizing the importance of clinical judgment in diagnosis.

The incidental finding of fibrous dysplasia of the left temporal bone was not believed to be related to the acute presentation. Fibrous dysplasia is a benign bone disorder caused by somatic mutations in the *GNAS *gene and typically presents with symptoms such as conductive hearing loss, facial asymmetry, or vertigo, depending on the involved structures [7,8]. In this case, no auditory or vestibular complaints were present, and the lesion did not contribute to the neurological findings.

This case underscores the primacy of clinical assessment in diagnosing WE, particularly when atypical features such as INO are present. Early administration of high-dose intravenous thiamine is the cornerstone of therapy and can prevent progression to irreversible complications like Korsakoff syndrome [5].

## Conclusions

WE remains a clinical diagnosis, particularly in non-alcoholic patients where imaging may not show classical features. This case illustrates a rare presentation of WE with INO and underscores the critical role of prompt, high-dose thiamine therapy. INO, though rare, can be an initial manifestation. Clinicians should maintain a high index of suspicion for WE in at-risk patients-even in the absence of typical signs-and initiate treatment without delay to avoid permanent neurological damage.
